# Supplementary material for: Phellinus baumii Polyphenol: A Potential Therapeutic Candidate against Lung Cancer Cells
Source: Int J Mol Sci. 2022 Dec 17;23(24):16141. doi: 10.3390/ijms232416141 (PMC9782521; doi:10.3390/ijms232416141)
Supplement: Supplementary file 1 [file ijms-23-16141-s001.zip › ijms-2094441-supplementary.pdf]

Supplementary File

Figure S1. Pathway with the largest proportion of genes enriched in KEGG: Pathway in cancer.

Figure S2. Histograms showing the expression levels of proteins involved in the regulation of apoptosis.

Figure S3. Histograms of the expression levels of cell cycle regulatory proteins quantified by western blotting analysis.

Table S1. Characterization of the chemical constituents of PBP by UPLC–ESI–QTOF–MS

Table S2. Potential targets of 17 compounds in PBP.

Table S3. The 60 potential targets of PBP for the treatment of lung cancer.

Table S4. GO enrichment analysis results.

Table S5. KEGG enrichment analysis results.

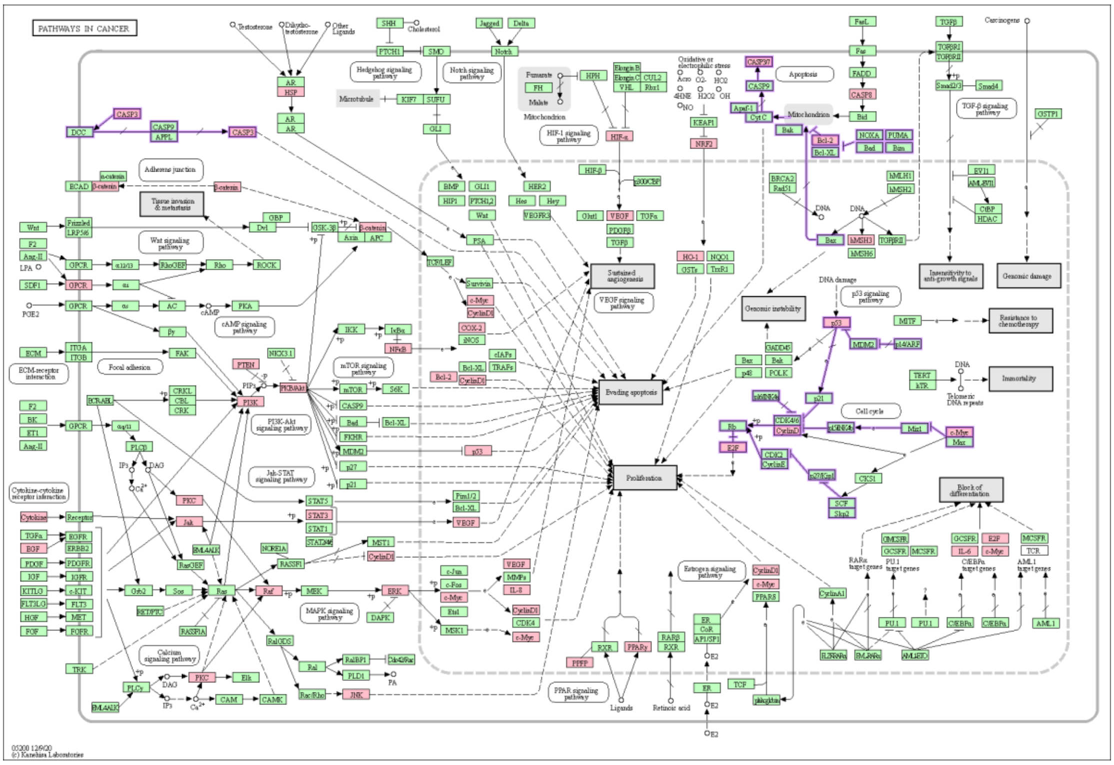

Figure S1. Pathway with the largest proportion of genes enriched in KEGG: Pathway in cancer.

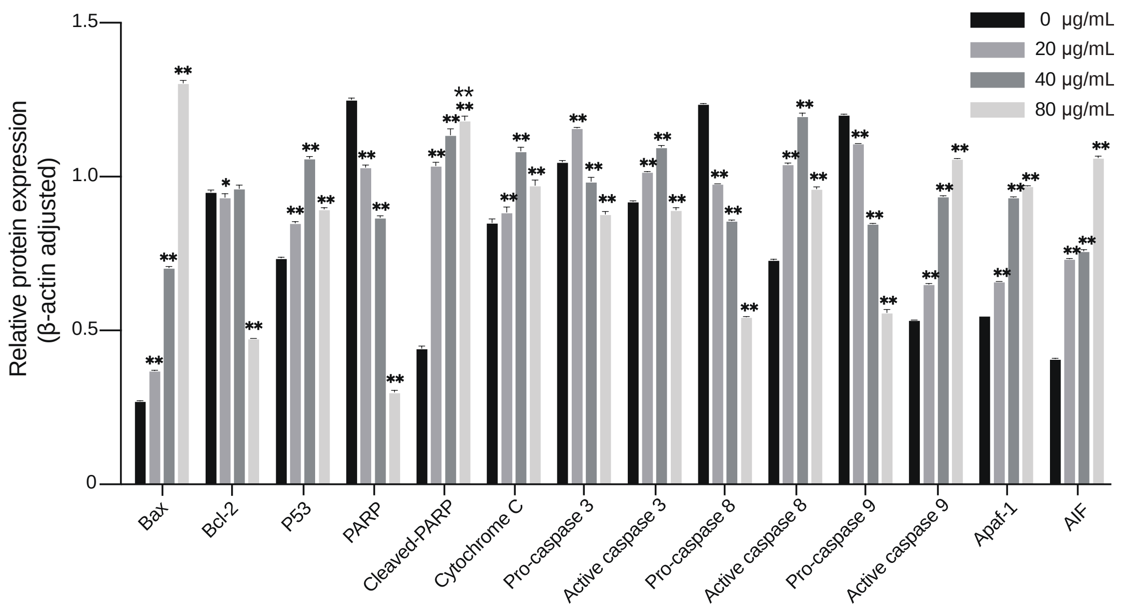

Figure S2. Histograms showing the expression levels of proteins involved in the regulation of apoptosis. The expression levels of the target proteins were normalized to those of β-actin.

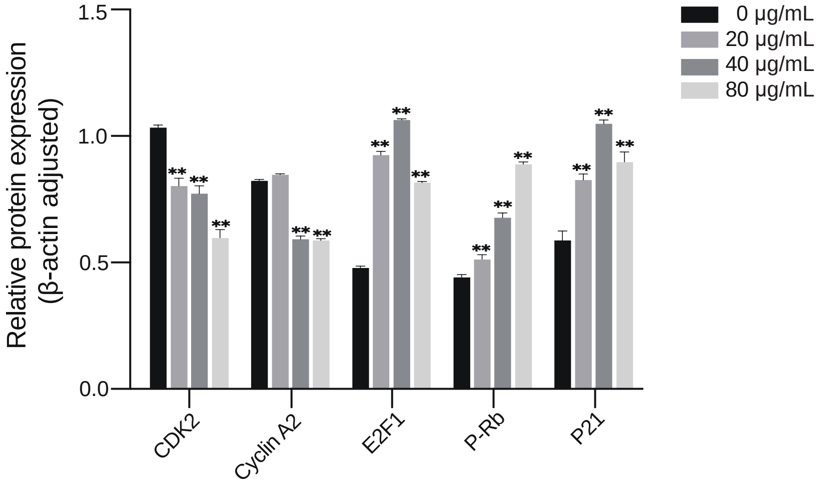

Figure S3. Histograms of the expression levels of cell cycle regulatory proteins quantified by western blotting analysis. The expression levels of the target proteins were normalized to those of β-actin.

Table S1. Characterization of the chemical constituents of PBP by UPLC–ESI–QTOF–MS

Peak No.	RT (min)	Formula	Negative Ion Mode				Indentification
			Theoretical Mass (m/z)	$[M-H]^-$ (m/z)	MS^2 (% base peak)	Mass Error (ppm)	
1	3.40	$C_7H_6O_3$	137.0244	137.0245	137.0220 (100), 91.0177 (17.78), 109.0271 (15.51), 81.0345 (14.02), 119.0110 (10.59), 65.0048 (7.04), 93.0336 (5.46)	0.2	protocatechuic aldehyde
2	3.67	$C_9H_8O_4$	179.0350	179.0347	135.0420 (100), 179.0306 (8.43), 89.0377 (5.63)	-1.8	caffeic acid
3	4.16	$C_{24}H_{20}O_8$	435.1085	435.1073	257.0421 (100), 255.0630 (94.67), 213.0524 (37.86), 177.0521 (25.34), 215.0310 (22.28), 435.1076 (22.08), 231.0623 (21.29), 173.0209 (16.79), 147.0418 (15.11), 417.0968 (10.97)	-3	kielcorin
4	4.40	$C_{22}H_{16}O_9$	423.0722	423.0705	159.0442 (100), 219.0266 (72.16), 175.0366 (62.82), 147.0418 (37.74), 243.0268 (36.83), 135.0425 (34.77), 199.0363 (33.39), 379.0812 (33.03), 335.0900 (32.35), 217.0470 (29.58)	-4	phellibaumin B
5	5.39	$C_{10}H_{10}O_3$	177.0557	177.0556	133.0274 (100), 177.0532 (53.08), 135.0429 (38.45), 161.0216 (18.19), 159.0425 (9.72)	-0.8	osmundacetone
6	5.86	$C_{26}H_{18}O_{10}$	489.0827	489.0815	241.0475 (100), 283.0585 (46.25), 199.0369 (45.23), 445.0924 (31.94), 335.0542 (30.50), 403.0810 (24.85), 309.0380 (23.52), 159.0422 (21.76), 323.0535 (16.02), 285.0374 (13.49)	-2.4	hypholomine B
7	5.73	$C_{13}H_{10}O_5$	245.0456	245.0445	159.0421 (100), 201.0524 (25.03), 135.0423 (13.85), 131.0472 (12.38), 141.0312 (6.56), 183.0417 (6.37)	-4.4	hispidin
8	6.21	$C_{23}H_{18}O_8$	421.0929	421.0917	217.0479 (100), 159.0424 (44.21), 241.0478 (13.46), 135.0427 (12.81), 203.0321 (11.07), 379.0812 (11.00), 175.0367 (10.28), 285.0387 (9.45), 243.0271 (9.06), 259.0589 (8.24)	-2.9	interfungin B

Peak No.	RT (min)	Formula	Negative Ion Mode				Indentification
			Theoretical Mass (m/z)	[M-H]- (m/z)	MS^2 (% base peak)	Mass Error (ppm)	
9	6.90	$C_{16}H_{14}O_6$	301.0718	301.0702	159.0425 (100), 135.0424 (22.41), 257.0791 (17.73), 301.0698 (16.80), 215.0685 (15.85), 213.0523 (8.73), 177.0527 (8.27), 131.0477 (7.83), 161.0209 (6.71), 185.0574 (6.41)	-5.1	sterubin
10	7.67	$C_{52}H_{32}O_{20}$	975.1414	975.1442	243.0262 (100), 199.0359 (85.20), 403.0448 (80.41), 175.0361 (78.13), 241.0466 (50.36), 487.0680 (27.88), 267.0260 (27.55), 443.0764 (27.32), 201.0144 (25.93), 239.0673 (24.15)	2.8	phelligridimer A
11	7.94	$C_{10}H_{10}O_2$	161.0608	161.0606	117.0325 (100), 161.0570 (43.69), 119.0471 (36.71)	-1	(E)-4-(4-hydroxyphenyl)-3-buten-2-one
12	8.43	$C_{25}H_{20}O_9$	463.1035	463.1021	259.0585 (100), 243.0270 (96.95), 159.0426 (90.26), 175.0370 (76.77), 217.0477 (74.85), 379.0812 (57.76), 135.0426 (37.49), 199.0370 (35.66), 335.0909 (31.27), 405.0605 (27.15)	-3	davallialactone
13	10.51	$C_{13}H_{14}O_5$	249.0769	249.0758	159.0422 (100), 161.0212 (87.03), 133.0271 (73.39), 249.0736 (24.73), 135.0424 (21.50), 177.0521 (14.31), 203.0318 (11.06), 131.0477 (10.95), 141.0314 (6.67)	-4.2	citrinin
14	11.31	$C_{24}H_{20}O_9$	451.1035	451.1018	243.0271 (100), 247.0586 (75.25), 405.0621 (32.55), 159.0423 (29.19), 135.0428 (19.85), 199.0373 (15.75), 175.0366 (13.57), 315.0492 (12.56), 451.1064 (12.30), 219.0270 (12.19)	-3.6	phellibaumin E
15	11.89	$C_{21}H_{14}O_9$	409.0565	409.0551	133.0267 (100), 205.0110 (61.18), 247.0216 (49.92), 177.0161 (41.59), 175.0367 (41.10), 159.0418 (40.28), 409.0549 (24.79), 219.0264 (24.32), 271.0217 (10.78), 109.0275 (7.89)	-3.5	3-(4,6-dihydroxy-2-oxo-2H-chromene-3-yl)-8-hydroxy-2-methoxy-2,3-dihydro-4H-furo[3,2-c]-chromene-4-one

Peak No.	RT (min)	Formula	Negative Ion Mode				Indentification
			Theoretical Mass (m/z)	[M-H]$^-$ (m/z)	MS^2 (% base peak)	Mass Error (ppm)	
16	13.13	$C_{33}H_{20}O_{13}$	623.0831	623.0832	419.0394 (100), 623.0871 (64.83), 417.0602 (26.95), 579.0962 (21.76), 469.0561 (13.36), 443.0399 (12.63), 373.0689 (12.41), 217.0100 (9.27), 375.0489 (8.63), 377.0281 (7.72)	0.2	phelligridin I
17	14.89	$C_{25}H_{18}O_9$	461.0878	461.0870	377.0661 (100), 159.0426 (60.47), 257.0432 (53.36), 461.0891 (42.68), 333.0754 (39.52), 241.0480 (32.25), 299.0542 (17.59), 213.0527 (15.38), 203.0321 (14.88), 305.0798 (12.25)	-1.7	inoscavin A

Table S2. Potential targets of 17 compounds in PBP.

Component name	Target name	Component name	Target name	Component name	Target name
protocatechuic aldehyde	TYR	osmundacetone	MAPK8	hispidin	CDC42
protocatechuic aldehyde	RB1	osmundacetone	GCLM	hispidin	IRS1
protocatechuic aldehyde	TNF	osmundacetone	SEC61B	hispidin	PTPRF
protocatechuic aldehyde	IL6	osmundacetone	HSPA5	hispidin	GCK
protocatechuic aldehyde	MAPK14	osmundacetone	KAT8	hispidin	FABP4
protocatechuic aldehyde	AKT1	osmundacetone	PIK3R6	hispidin	GLP1R
protocatechuic aldehyde	CTNNB1	osmundacetone	TYR	hispidin	ATP5I
protocatechuic aldehyde	BCL2	osmundacetone	CPLX2	hispidin	CDK2
protocatechuic aldehyde	PTGS2	osmundacetone	GORASP1	hispidin	PPARGC1A
protocatechuic aldehyde	CDH1	osmundacetone	AIMP2	hispidin	ADIG
protocatechuic aldehyde	CCND1	osmundacetone	RAX	hispidin	RUNX2
protocatechuic aldehyde	CASP3	osmundacetone	HYOU1	sterubin	EPHB2
protocatechuic aldehyde	AKR1B1	osmundacetone	CCNE2	sterubin	MITF
protocatechuic aldehyde	PIK3CA	osmundacetone	PIK3CA	sterubin	CACNA1C
protocatechuic aldehyde	GAA	osmundacetone	GLUD1	sterubin	TYR
protocatechuic aldehyde	DCT	osmundacetone	CCNA2	sterubin	GABPA
protocatechuic aldehyde	NFE2L2	osmundacetone	PDXK	phelligridimer A	PTPN1
protocatechuic aldehyde	PARK7	osmundacetone	DNASE1	phelligridimer A	AKR1B1
protocatechuic aldehyde	NOS3	osmundacetone	CAT	phelligridimer A	PTPRF
protocatechuic aldehyde	AGER	osmundacetone	MBL2	phelligridimer A	F2
protocatechuic aldehyde	HDAC2	osmundacetone	DDIT3	phelligridimer A	IL6
protocatechuic aldehyde	CCK	osmundacetone	GCLC	phelligridimer A	INS
protocatechuic aldehyde	NOX4	osmundacetone	PMEL	(E)-4-(4-hydroxyphenyl)-3-buten-2-one	TUBB4B
protocatechuic aldehyde	CYP2D6	osmundacetone	TYRP1	(E)-4-(4-hydroxyphenyl)-3-buten-3-one	IFNLR1
protocatechuic aldehyde	VEGFA	osmundacetone	PLK1	(E)-4-(4-hydroxyphenyl)-3-buten-4-one	IL6
protocatechuic aldehyde	CYP1A2	osmundacetone	ATF6	davallialactone	MAPK1
protocatechuic aldehyde	CXCR4	osmundacetone	SIRT3	davallialactone	F2RL2
protocatechuic aldehyde	HMGB1	osmundacetone	TNF	davallialactone	PTGS2
protocatechuic aldehyde	IL13	osmundacetone	IL6	davallialactone	F2R
protocatechuic aldehyde	PECAM1	osmundacetone	GSK3B	davallialactone	ADH5
protocatechuic aldehyde	CAT	osmundacetone	GLS	davallialactone	AKR1B1
protocatechuic aldehyde	JAK2	osmundacetone	NQO1	davallialactone	PTPRF
protocatechuic aldehyde	KDR	osmundacetone	PXN	davallialactone	VCAM1
protocatechuic aldehyde	CD40	osmundacetone	NFKBIA	davallialactone	FASN
protocatechuic aldehyde	MAPK3	osmundacetone	CDKN1B	davallialactone	GAA
protocatechuic aldehyde	SNCA	osmundacetone	PTPRF	davallialactone	CD14
protocatechuic aldehyde	SIRT1	osmundacetone	SLC2A4	davallialactone	ICAM1

protocatechuic aldehyde	HIF1A	osmundacetone	RUNX2	davallialactone	PARP1
protocatechuic aldehyde	PTEN	osmundacetone	MSMP	davallialactone	ELANE
protocatechuic aldehyde	ALB	osmundacetone	IFNG	davallialactone	INS
protocatechuic aldehyde	CASP9	osmundacetone	PTK2	davallialactone	HMOX1
protocatechuic aldehyde	PTP4A1	osmundacetone	GAA	davallialactone	NOS2
protocatechuic aldehyde	JUND	osmundacetone	IL1B	davallialactone	TLR4
protocatechuic aldehyde	NOMO1	osmundacetone	GABPA	davallialactone	MAPK8
protocatechuic aldehyde	DEFB4A	osmundacetone	MYC	davallialactone	ITGAM
protocatechuic aldehyde	VIM	osmundacetone	CCND1	davallialactone	F2
protocatechuic aldehyde	PPARG	osmundacetone	LDHB	davallialactone	PIK3CA
protocatechuic aldehyde	HMOX1	osmundacetone	BGLAP	davallialactone	GPT
protocatechuic aldehyde	STAT3	osmundacetone	MYLK	davallialactone	MAPK14
protocatechuic aldehyde	CDH3	osmundacetone	LYZ	davallialactone	IL6
protocatechuic aldehyde	ANXA5	osmundacetone	JUN	davallialactone	AKT1
protocatechuic aldehyde	PDZK1	osmundacetone	LIPE	citrinin	PDS5B
protocatechuic aldehyde	PPP1R3A	osmundacetone	TLR4	citrinin	CASP3
protocatechuic aldehyde	MAPK8	osmundacetone	MPO	citrinin	CAT
protocatechuic aldehyde	CA1	osmundacetone	ABCB1	citrinin	SPATA5
protocatechuic aldehyde	NEK7	osmundacetone	EGF	citrinin	PIGR
protocatechuic aldehyde	ANG	osmundacetone	ACE	citrinin	CUBN
protocatechuic aldehyde	MRPS28	osmundacetone	PTGS2	citrinin	CASP9
protocatechuic aldehyde	ODC1	osmundacetone	GCG	citrinin	HSD17B6
protocatechuic aldehyde	CHRNA7	osmundacetone	CASP3	citrinin	BCL2
protocatechuic aldehyde	CHRM3	osmundacetone	SERPINB10	citrinin	LACE1
protocatechuic aldehyde	CHRM2	osmundacetone	INS	citrinin	HMGCR
protocatechuic aldehyde	CHRM1	hypholomine B	AKR1B1	citrinin	ABCC3
protocatechuic aldehyde	LYZD1	hypholomine B	PTPRF	citrinin	KIF14
protocatechuic aldehyde	GABRA6	hypholomine B	SREBF1	citrinin	TNF
protocatechuic aldehyde	GABRA3	hypholomine B	GCK	citrinin	CD69
protocatechuic aldehyde	GABRA2	hypholomine B	PPARGC1A	citrinin	MAPK8
protocatechuic aldehyde	GABRA1	hypholomine B	FASN	citrinin	HSPA5
protocatechuic aldehyde	ADH1C	hypholomine B	GAA	citrinin	GPT
caffeic acid	CAT	hypholomine B	SIRT1	citrinin	IL10
caffeic acid	MGAM	hypholomine B	PRKAA2	citrinin	PAK2
caffeic acid	TYR	hypholomine B	INS	citrinin	PPP1R15A
caffeic acid	ALOX5	hypholomine B	ADIPOQ	citrinin	PDAP1
caffeic acid	TNF	hypholomine B	PPARG	citrinin	TFDP3
caffeic acid	PTGS2	hypholomine B	IL6	citrinin	TFPT
caffeic acid	IL6	hispidin	PRKCQ	citrinin	POLR2B
caffeic acid	COMT	hispidin	PRKCD	citrinin	TP53
caffeic acid	XDH	hispidin	PRKX	citrinin	ATF1
caffeic acid	ACHE	hispidin	PRKCB	citrinin	LAMP2
caffeic acid	NFE2L2	hispidin	PAK1	citrinin	SLC22A8

caffeic acid	AKT1	hispidin	CASP3	citrinin	LDHB
caffeic acid	HMOX1	hispidin	AKR1B1	citrinin	SLC22A6
caffeic acid	CASP3	hispidin	PRKAA2	citrinin	XRCC6
caffeic acid	BCHE	hispidin	OLR1	citrinin	JUN
caffeic acid	HPGDS	hispidin	HMOX1	citrinin	RYR1
caffeic acid	NOS2	hispidin	AKT1	citrinin	XBP1
caffeic acid	INS	hispidin	PRKCE	citrinin	OGG1
caffeic acid	MPO	hispidin	PRKCA	citrinin	IL6
caffeic acid	BCL2	hispidin	BACE1	citrinin	PROK1
caffeic acid	MAPK14	hispidin	PIK3CA	citrinin	CCNB1
caffeic acid	LIPE	hispidin	INS	citrinin	RAF1
caffeic acid	CXCL8	hispidin	SREBF1	citrinin	ATP5I
caffeic acid	MAPK8	hispidin	VCAM1	citrinin	HSP90AA1
caffeic acid	CYP1B1	hispidin	CACNA1C	citrinin	FASN
caffeic acid	GSR	hispidin	CAT	citrinin	TMEM199
caffeic acid	GPT	hispidin	GAA	citrinin	TXN
caffeic acid	CCL2	hispidin	SIRT1	citrinin	SOD2
caffeic acid	VEGFA	hispidin	TYR	citrinin	MAML3
caffeic acid	ANXA5	hispidin	ELANE	citrinin	CASP8
caffeic acid	GORASP1	hispidin	XDH	citrinin	ENPP2
caffeic acid	PIK3CA	hispidin	CASP9	citrinin	PMPCB
caffeic acid	TP53	hispidin	TUBB4B	citrinin	KCNQ2
caffeic acid	IL10	hispidin	PPARG	citrinin	G6PD
caffeic acid	LDHB	hispidin	DSC2	citrinin	ALB
caffeic acid	ELANE	hispidin	STAT3	citrinin	FBXL5
caffeic acid	JUN	hispidin	RGS10	citrinin	GSR
caffeic acid	ANG	hispidin	PLCB2	citrinin	SLC22A10
caffeic acid	MAGEE1	hispidin	NOS2	citrinin	FBXO38
caffeic acid	NFKB1	hispidin	CACNA1I	citrinin	TUBB4B
caffeic acid	ARG1	hispidin	PAK4	citrinin	SCGB2A2
caffeic acid	AKR1B1	hispidin	PTGS2	citrinin	MKL1
caffeic acid	PNLIP	hispidin	LMNB1	citrinin	OPA3
caffeic acid	ACE	hispidin	RGS2	citrinin	LIPE
caffeic acid	BDNF	hispidin	RGS4	citrinin	CD79A
caffeic acid	ALB	hispidin	DSG1	citrinin	PDCD10
caffeic acid	PTGS1	hispidin	LPAR1	citrinin	MAP3K13
caffeic acid	MSMP	hispidin	EARS2	citrinin	PDIA6
caffeic acid	STAT3	hispidin	ITGA4	citrinin	AIMP2
caffeic acid	PPARG	hispidin	NCF2	citrinin	LMCD1
caffeic acid	IL4	hispidin	CASP6	citrinin	HPGDS
caffeic acid	FASN	hispidin	SERPINB10	citrinin	ABCB1
caffeic acid	NOS3	hispidin	PREP	citrinin	CCNG1
caffeic acid	SLC2A2	hispidin	PDIA3	citrinin	SLC22A11

caffeic acid	HMGCR	hispidin	STK11	citrinin	CXCL8
caffeic acid	IL17A	hispidin	SLC18A2	citrinin	CPA6
caffeic acid	CASP9	hispidin	PTPN1	citrinin	RAB11A
caffeic acid	GBA	hispidin	LAMP1	phellibaumin E	NOMO1
caffeic acid	SREBF1	hispidin	GCLC	phellibaumin E	CAPN3
caffeic acid	SOD2	hispidin	BMP7	phellibaumin E	KLHDC8B
caffeic acid	MB	hispidin	E2F1	inoscavin A	NOMO1
caffeic acid	ADIPOQ	hispidin	MMP3	inoscavin A	PDZK1
caffeic acid	IL1B	hispidin	FOXO3	inoscavin A	ETF1
caffeic acid	CASP8	hispidin	MSH3	inoscavin A	CAPN3
caffeic acid	HIF1A	hispidin	FNTA	inoscavin A	PTCH1
caffeic acid	ADRB1	hispidin	SP7	inoscavin A	KLHDC8B
caffeic acid	ADRA2A	hispidin	GCLM	inoscavin A	GLI1
caffeic acid	ADRA2C	hispidin	PNLIP	inoscavin A	AKR1B1
caffeic acid	ADRB2	hispidin	SHC1	inoscavin A	PTPRF
caffeic acid	MAOB	hispidin	SGCB	inoscavin A	GAA
caffeic acid	MAOA	hispidin	JAK1	inoscavin A	INS
caffeic acid	CTRB1	hispidin	ATG5	inoscavin A	ABCB1
osmundacetone	HMOX1	hispidin	PTK2	inoscavin A	IL6
osmundacetone	AKT1	hispidin	NPPB	Kielcorin	-
osmundacetone	MAPK8	hispidin	ITGB1	phellibaumin B	-
interfungin B	-	phelligridin I	-	3-(4,6-Dihydroxy-2-oxochromen-3-yl)-8-hydroxy-2-methoxy-2,3-dihydrofuro [3,2-c] chromen-4-one	-

Table S3. The 60 potential targets of PBP for the treatment of lung cancer.

NCBI Gene ID	Full Name	Symbol
5925	Retinoblastoma-associated protein	RB1
7124	Tumor necrosis factor	TNF
3569	Interleukin-6	IL6
1432	Mitogen-activated protein kinase 14	MAPK14
207	RAC-alpha serine/threonine-protein kinase	AKT1
1499	Catenin beta-1	CTNNB1
596	Apoptosis regulator Bcl-2	BCL2
5743	Prostaglandin G/H synthase 2	PTGS2
999	Cadherin-1	CDH1
595	G1/S-specific cyclin-D1	CCND1
836	Caspase-3	CASP3

5290	Phosphatidylinositol 4,5-bisphosphate 3-kinase catalytic subunit alpha isoform	PIK3CA
11315	Protein/nucleic acid deglycase DJ-1	PARK7
7422	Vascular endothelial growth factor A	VEGFA
1544	Cytochrome p450 family 1 subfamily a polypeptide 2	CYP1A2
3791	Vascular endothelial growth factor receptor 2	KDR
5595	Mitogen-activated protein kinase 3	MAPK3
3091	Hypoxia-inducible factor 1-alpha	HIF1A
5728	Phosphatase and tensin homolog	PTEN
3727	Transcription factor jun-D	JUND
5468	Peroxisome proliferator-activated receptor gamma	PPARG
3162	Heme oxygenase 1	HMOX1
6774	Signal transducer and activator of transcription 3	STAT3
1139	Neuronal acetylcholine receptor subunit alpha-7	CHRNA7
4843	Nitric-oxide synthase, inducible	NOS2
4353	Myeloperoxidase	MPO
3576	Interleukin-8	CXCL8
1545	Cytochrome p450 family 1 subfamily b polypeptide 1	CYP1B1
7157	Cellular tumor antigen p53	TP53
3586	Interleukin-10	IL10
3725	Transcription factor AP-1	JUN
1636	Angiotensin-converting enzyme	ACE
3553	Interleukin-1 beta	IL1B
841	Caspase-8	CASP8
2730	Glutamate--cysteine ligase regulatory subunit	GCLM
2729	Glutamate--cysteine ligase catalytic subunit	GCLC
7306	5,6-dihydroxyindole-2-carboxylic acid oxidase	TYRP1
1027	Cyclin-dependent kinase inhibitor 1B	CDKN1B
3458	Interferon gamma	IFNG
4609	Myc proto-oncogene protein	MYC
7099	Toll-like receptor 4	TLR4
5243	Multidrug resistance protein 1	ABCB1
1950	Pro-epidermal growth factor	EGF
5550	Prolyl endopeptidase	PREP
6794	Serine/threonine-protein kinase STK11	STK11
4314	Matrix metalloproteinase-3 (stromelysin 1, progelatinase)	MMP3
4437	DNA mismatch repair protein Msh3	MSH3
3688	Integrin beta-1	ITGB1
998	Cell division control protein 42 homolog	CDC42
1017	Cyclin-dependent kinase 2	CDK2
2048	Ephrin type-B receptor 2	EPHB2
163702	Interferon lambda receptor 1	IFNLR1
5594	Mitogen-activated protein kinase 1	MAPK1

4968	N-glycosylase/DNA lyase	OGG1
5894	RAF proto-oncogene serine/threonine-protein kinase	RAF1
900	Cyclin-G1	CCNG1
5727	Protein patched homolog 1	PTCH1
142	Poly [ADP-ribose] polymerase 1	PARP1
842	Caspase-9	CASP9
1869	Transcription factor E2F1	E2F1

Table S4. GO enrichment analysis results.

Category	Term	Count	%	P Value
GOTERM_BP	response to drug	19	32.20	2.73E-19
GOTERM_BP	response to xenobiotic stimulus	18	30.51	2.79E-19
GOTERM_BP	response to estradiol	14	23.73	1.03E-17
GOTERM_BP	positive regulation of gene expression	21	35.59	1.65E-17
GOTERM_BP	aging	15	25.42	4.46E-16
GOTERM_BP	negative regulation of gene expression	16	27.12	1.32E-14
GOTERM_BP	negative regulation of apoptotic process	18	30.51	1.37E-13
GOTERM_BP	cellular response to hypoxia	12	20.34	4.21E-13
GOTERM_BP	angiogenesis	14	23.73	4.38E-13
GOTERM_BP	positive regulation of apoptotic process	15	25.42	6.54E-13
GOTERM_BP	response to lipopolysaccharide	12	20.34	1.18E-12
GOTERM_BP	regulation of cell cycle	14	23.73	1.05E-11
GOTERM_BP	negative regulation of neuron apoptotic process	11	18.64	3.76E-11
GOTERM_BP	negative regulation of cell proliferation	15	25.42	6.81E-11
GOTERM_BP	positive regulation of pri-miRNA transcription from RNA polymerase II promoter	8	13.56	1.02E-10
GOTERM_BP	positive regulation of transcription from RNA polymerase II promoter	21	35.59	1.28E-10
GOTERM_BP	positive regulation of transcription, DNA-templated	17	28.81	2.43E-10
GOTERM_BP	positive regulation of peptidyl-serine phosphorylation	9	15.25	2.81E-10
GOTERM_BP	response to activity	8	13.56	4.05E-10
GOTERM_BP	cellular response to DNA damage stimulus	12	20.34	5.66E-10
GOTERM_BP	positive regulation of angiogenesis	10	16.95	1.38E-09
GOTERM_BP	positive regulation of smooth muscle cell proliferation	8	13.56	1.57E-09
GOTERM_BP	lipopolysaccharide-mediated signaling pathway	7	11.86	1.70E-09
GOTERM_BP	cellular response to cadmium ion	7	11.86	2.33E-09
GOTERM_BP	positive regulation of sequence-specific DNA binding transcription factor activity	9	15.25	2.43E-09
GOTERM_BP	liver regeneration	7	11.86	2.72E-09
GOTERM_BP	response to oxidative stress	9	15.25	2.78E-09
GOTERM_BP	response to hypoxia	10	16.95	3.45E-09
GOTERM_BP	positive regulation of cell proliferation	14	23.73	6.89E-09
GOTERM_BP	cellular response to organic cyclic compound	7	11.86	1.46E-08

GOTERM_BP	apoptotic process	14	23.73	2.22E-08
GOTERM_BP	positive regulation of MAPK cascade	9	15.25	2.52E-08
GOTERM_BP	positive regulation of neuron apoptotic process	7	11.86	3.35E-08
GOTERM_BP	positive regulation of interleukin-8 production	7	11.86	4.06E-08
GOTERM_BP	glucose homeostasis	8	13.56	4.87E-08
GOTERM_BP	cellular response to lipopolysaccharide	9	15.25	1.08E-07
GOTERM_BP	positive regulation of endothelial cell proliferation	7	11.86	1.13E-07
GOTERM_BP	epithelial cell apoptotic process	5	8.47	1.30E-07
GOTERM_BP	positive regulation of protein phosphorylation	9	15.25	2.06E-07
GOTERM_BP	positive regulation of chemokine production	6	10.17	2.29E-07
GOTERM_BP	cellular response to mechanical stimulus	7	11.86	2.36E-07
GOTERM_BP	positive regulation of nitric oxide biosynthetic process	6	10.17	3.53E-07
GOTERM_BP	protein kinase B signaling	6	10.17	5.27E-07
GOTERM_BP	ERK1 and ERK2 cascade	6	10.17	6.36E-07
GOTERM_BP	positive regulation of interleukin-6 production	7	11.86	6.43E-07
GOTERM_BP	negative regulation of transcription from RNA polymerase II promoter	15	25.42	7.50E-07
GOTERM_BP	macrophage differentiation	5	8.47	8.85E-07
GOTERM_BP	vascular endothelial growth factor production	4	6.78	8.93E-07
GOTERM_BP	cellular response to insulin stimulus	7	11.86	8.98E-07
GOTERM_BP	positive regulation of interleukin-1 beta production	6	10.17	1.48E-06
GOTERM_BP	positive regulation of cytokine production involved in inflammatory response	5	8.47	1.65E-06
GOTERM_BP	response to glucocorticoid	6	10.17	1.72E-06
GOTERM_BP	positive regulation of vascular endothelial growth factor production	5	8.47	1.89E-06
GOTERM_BP	G1/S transition of mitotic cell cycle	6	10.17	2.82E-06
GOTERM_BP	neuron apoptotic process	6	10.17	2.82E-06
GOTERM_BP	inflammatory response	10	16.95	3.55E-06
GOTERM_BP	positive regulation of ERK1 and ERK2 cascade	8	13.56	5.42E-06
GOTERM_BP	positive regulation of receptor activity	4	6.78	5.55E-06
GOTERM_BP	response to nicotine	5	8.47	6.18E-06
GOTERM_BP	positive regulation of protein import into nucleus	5	8.47	9.10E-06
GOTERM_BP	negative regulation of vascular smooth muscle cell proliferation	5	8.47	9.96E-06
GOTERM_BP	regulation of gene expression	8	13.56	1.09E-05
GOTERM_BP	positive regulation of cell migration	8	13.56	1.18E-05
GOTERM_BP	regulation of insulin secretion	5	8.47	1.19E-05
GOTERM_BP	thymus development	5	8.47	1.29E-05
GOTERM_BP	striated muscle cell differentiation	4	6.78	1.40E-05
GOTERM_BP	stem cell proliferation	5	8.47	1.78E-05
GOTERM_BP	positive regulation of protein complex assembly	5	8.47	2.23E-05
GOTERM_BP	positive regulation of DNA biosynthetic process	4	6.78	2.41E-05

GOTERM_BP	positive regulation of protein localization to plasma membrane	5	8.47	2.57E-05
GOTERM_BP	positive regulation of calcidiol 1-monooxygenase activity	3	5.08	2.66E-05
GOTERM_BP	response to muscle stretch	4	6.78	2.83E-05
GOTERM_BP	cellular response to UV	5	8.47	2.95E-05
GOTERM_BP	positive regulation of beta-amyloid formation	4	6.78	3.29E-05
GOTERM_BP	cell migration involved in sprouting angiogenesis	4	6.78	3.29E-05
GOTERM_BP	nitric oxide biosynthetic process	4	6.78	3.80E-05
GOTERM_BP	negative regulation of cell growth	6	10.17	3.94E-05
GOTERM_BP	MAPK cascade	6	10.17	4.09E-05
GOTERM_BP	positive regulation of protein kinase B signaling	6	10.17	4.25E-05
GOTERM_BP	cellular response to xenobiotic stimulus	5	8.47	4.33E-05
GOTERM_BP	response to mechanical stimulus	5	8.47	4.33E-05
GOTERM_BP	positive regulation of glial cell proliferation	4	6.78	4.37E-05
GOTERM_BP	learning or memory	5	8.47	4.60E-05
GOTERM_BP	glucose metabolic process	5	8.47	4.88E-05
GOTERM_BP	positive regulation of smooth muscle cell migration	4	6.78	4.98E-05
GOTERM_BP	glial cell apoptotic process	3	5.08	5.30E-05
GOTERM_BP	response to human chorionic gonadotropin	3	5.08	5.30E-05
GOTERM_BP	response to ethanol	6	10.17	5.47E-05
GOTERM_BP	negative regulation of cysteine-type endopeptidase activity involved in apoptotic process	5	8.47	5.48E-05
GOTERM_BP	execution phase of apoptosis	4	6.78	5.64E-05
GOTERM_BP	cellular response to tumor necrosis factor	6	10.17	7.66E-05
GOTERM_BP	positive regulation of neuroblast proliferation	4	6.78	7.99E-05
GOTERM_BP	response to nutrient	5	8.47	8.00E-05
GOTERM_BP	transcription from RNA polymerase II promoter	7	11.86	8.75E-05
GOTERM_BP	positive regulation of fever generation	3	5.08	8.82E-05
GOTERM_BP	decidualization	4	6.78	8.89E-05
GOTERM_BP	response to gamma radiation	4	6.78	8.89E-05
GOTERM_BP	positive regulation of MAP kinase activity	5	8.47	9.78E-05
GOTERM_BP	response to radiation	4	6.78	1.09E-04
GOTERM_BP	positive regulation of translation	5	8.47	1.18E-04
GOTERM_BP	response to antibiotic	4	6.78	1.20E-04
GOTERM_BP	cellular response to virus	5	8.47	1.30E-04
GOTERM_BP	cellular response to fibroblast growth factor stimulus	4	6.78	1.32E-04
GOTERM_BP	positive regulation of protein localization to nucleus	4	6.78	1.32E-04
GOTERM_BP	negative regulation of apoptotic signaling pathway	4	6.78	1.32E-04
GOTERM_BP	response to cobalt ion	3	5.08	1.32E-04
GOTERM_BP	positive regulation of transcription from RNA polymerase II promoter in response to hypoxia	3	5.08	1.32E-04

GOTERM_BP	response to toxic substance	5	8.47	1.36E-04
GOTERM_BP	positive regulation of telomerase activity	4	6.78	1.44E-04
GOTERM_BP	cellular response to vascular endothelial growth factor stimulus	4	6.78	1.44E-04
GOTERM_BP	Ras protein signal transduction	5	8.47	1.55E-04
GOTERM_BP	positive regulation of cell migration involved in sprouting angiogenesis	4	6.78	1.57E-04
GOTERM_BP	neuroblast proliferation	4	6.78	1.71E-04
GOTERM_BP	trachea formation	3	5.08	1.84E-04
GOTERM_BP	positive regulation of JAK-STAT cascade	4	6.78	1.86E-04
GOTERM_BP	cellular response to reactive oxygen species	4	6.78	2.01E-04
GOTERM_BP	negative regulation of extrinsic apoptotic signaling pathway in absence of ligand	4	6.78	2.01E-04
GOTERM_BP	peptidyl-serine phosphorylation	6	10.17	2.10E-04
GOTERM_BP	extrinsic apoptotic signaling pathway via death domain receptors	4	6.78	2.18E-04
GOTERM_BP	cellular response to interferon-gamma	5	8.47	2.23E-04
GOTERM_BP	epithelial cell proliferation involved in prostate gland development	3	5.08	2.45E-04
GOTERM_BP	positive regulation of I-kappaB kinase/NF-kappaB signaling	6	10.17	2.76E-04
GOTERM_BP	positive regulation of tumor necrosis factor production	5	8.47	3.11E-04
GOTERM_BP	branching involved in ureteric bud morphogenesis	4	6.78	3.12E-04
GOTERM_BP	ovarian follicle development	4	6.78	3.12E-04
GOTERM_BP	signal transduction	13	22.03	3.22E-04
GOTERM_BP	positive regulation of inflammatory response	5	8.47	3.34E-04
GOTERM_BP	negative regulation of transcription, DNA-templated	9	15.25	3.62E-04
GOTERM_BP	cellular response to beta-amyloid	4	6.78	3.80E-04
GOTERM_BP	negative regulation of lipid storage	3	5.08	3.93E-04
GOTERM_BP	fibroblast apoptotic process	3	5.08	3.93E-04
GOTERM_BP	in utero embryonic development	6	10.17	4.44E-04
GOTERM_BP	intrinsic apoptotic signaling pathway in response to DNA damage	4	6.78	4.56E-04
GOTERM_BP	positive regulation of blood vessel endothelial cell migration	4	6.78	4.56E-04
GOTERM_BP	commissural neuron axon guidance	3	5.08	4.79E-04
GOTERM_BP	heart development	6	10.17	5.04E-04
GOTERM_BP	chondrocyte differentiation	4	6.78	5.42E-04
GOTERM_BP	response to hydrogen peroxide	4	6.78	5.42E-04
GOTERM_BP	cellular senescence	4	6.78	5.72E-04
GOTERM_BP	negative regulation of autophagy	4	6.78	5.72E-04
GOTERM_BP	anoikis	3	5.08	5.74E-04
GOTERM_BP	sensory perception of pain	4	6.78	6.04E-04
GOTERM_BP	phosphorylation	5	8.47	6.28E-04
GOTERM_BP	mitotic G1 DNA damage checkpoint	3	5.08	6.77E-04

GOTERM_BP	protein phosphorylation	8	13.56	7.27E-04
GOTERM_BP	I-kappaB kinase/NF-kappaB signaling	4	6.78	8.18E-04
GOTERM_BP	humoral immune response	4	6.78	8.18E-04
GOTERM_BP	negative regulation of epithelial cell apoptotic process	3	5.08	9.08E-04
GOTERM_BP	positive regulation of transcription regulatory region DNA binding	3	5.08	9.08E-04
GOTERM_BP	response to arsenic-containing substance	3	5.08	9.08E-04
GOTERM_BP	positive regulation of MHC class II biosynthetic process	3	5.08	9.08E-04
GOTERM_BP	negative regulation of macroautophagy	3	5.08	9.08E-04
GOTERM_BP	response to ischemia	4	6.78	9.41E-04
GOTERM_BP	positive regulation of heterotypic cell-cell adhesion	3	5.08	0.00103581
GOTERM_BP	regulation of ossification	3	5.08	0.00103581
GOTERM_BP	vascular endothelial growth factor signaling pathway	3	5.08	0.00103581
GOTERM_BP	cellular response to hepatocyte growth factor stimulus	3	5.08	0.00103581
GOTERM_BP	cellular response to drug	4	6.78	0.00107514
GOTERM_BP	positive regulation of endothelial cell migration	4	6.78	0.00112238
GOTERM_BP	negative regulation of glucose import	3	5.08	0.00117165
GOTERM_BP	astrocyte activation	3	5.08	0.00117165
GOTERM_BP	response to dexamethasone	3	5.08	0.00117165
GOTERM_BP	circadian rhythm	4	6.78	0.00122074
GOTERM_BP	negative regulation of protein kinase activity	4	6.78	0.00122074
GOTERM_BP	cytokine-mediated signaling pathway	5	8.47	0.00123937
GOTERM_BP	response to estrogen	4	6.78	0.00127189
GOTERM_BP	peptidyl-threonine phosphorylation	4	6.78	0.00127189
GOTERM_BP	mammary gland alveolus development	3	5.08	0.00131557
GOTERM_BP	positive regulation of membrane protein ectodomain proteolysis	3	5.08	0.00131557
GOTERM_BP	positive regulation of nitric-oxide synthase biosynthetic process	3	5.08	0.00131557
GOTERM_BP	response to iron ion	3	5.08	0.0014675
GOTERM_BP	negative regulation of anoikis	3	5.08	0.0014675
GOTERM_BP	monocyte differentiation	3	5.08	0.0014675
GOTERM_BP	dendritic spine morphogenesis	3	5.08	0.0014675
GOTERM_BP	regulation of cell proliferation	5	8.47	0.00149
GOTERM_BP	positive regulation of macrophage activation	3	5.08	0.00162742
GOTERM_BP	negative regulation of B cell proliferation	3	5.08	0.00162742
GOTERM_BP	response to X-ray	3	5.08	0.00162742
GOTERM_BP	regulation of blood pressure	4	6.78	0.00179373
GOTERM_BP	face development	3	5.08	0.00179526
GOTERM_BP	hematopoietic stem cell differentiation	3	5.08	0.00179526
GOTERM_BP	activation of cysteine-type endopeptidase activity involved in apoptotic process	4	6.78	0.00192527

GOTERM_BP	release of cytochrome c from mitochondria	3	5.08	0.00197098
GOTERM_BP	positive regulation of phosphatidylinositol 3-kinase signaling	4	6.78	0.00199324
GOTERM_BP	positive regulation of mesenchymal cell proliferation	3	5.08	0.00215454
GOTERM_BP	cellular response to glucose stimulus	4	6.78	0.00220605
GOTERM_BP	intracellular signal transduction	7	11.86	0.00227828
GOTERM_BP	positive regulation of glycolytic process	3	5.08	0.00234588
GOTERM_BP	negative regulation of neurogenesis	3	5.08	0.00234588
GOTERM_BP	negative regulation of reactive oxygen species metabolic process	3	5.08	0.00234588
GOTERM_BP	cell proliferation	5	8.47	0.00235812
GOTERM_BP	positive regulation of nitric-oxide synthase activity	3	5.08	0.00275173
GOTERM_BP	regulation of protein stability	4	6.78	0.00275605
GOTERM_BP	positive regulation of immunoglobulin production	3	5.08	0.00296615
GOTERM_BP	adherens junction organization	3	5.08	0.00296615
GOTERM_BP	thyroid gland development	3	5.08	0.00318817
GOTERM_BP	regulation of blood vessel diameter	3	5.08	0.00318817
GOTERM_BP	positive regulation of JNK cascade	4	6.78	0.00319628
GOTERM_BP	transforming growth factor beta receptor signaling pathway	4	6.78	0.00347996
GOTERM_BP	microglial cell activation	3	5.08	0.00365483
GOTERM_BP	positive regulation of mitotic nuclear division	3	5.08	0.00365483
GOTERM_BP	vascular endothelial growth factor receptor signaling pathway	3	5.08	0.00365483
GOTERM_BP	maintenance of permeability of blood-brain barrier	3	5.08	0.00389938
GOTERM_BP	positive regulation of DNA replication	3	5.08	0.00415134
GOTERM_BP	branching involved in blood vessel morphogenesis	3	5.08	0.00415134
GOTERM_BP	positive regulation of macroautophagy	3	5.08	0.00415134
GOTERM_BP	negative regulation of cyclin-dependent protein serine/threonine kinase activity	3	5.08	0.00441067
GOTERM_BP	positive regulation of DNA binding	3	5.08	0.00441067
GOTERM_BP	positive regulation of erythrocyte differentiation	3	5.08	0.00467734
GOTERM_BP	positive regulation of telomere maintenance via telomerase	3	5.08	0.00467734
GOTERM_BP	endothelial cell migration	3	5.08	0.00467734
GOTERM_BP	negative regulation of mitotic cell cycle	3	5.08	0.00495128
GOTERM_BP	positive regulation of reactive oxygen species metabolic process	3	5.08	0.00495128
GOTERM_BP	response to tumor necrosis factor	3	5.08	0.00495128
GOTERM_CC	nucleus	34	57.63	6.67E-10
GOTERM_CC	cytosol	33	55.93	3.08E-08
GOTERM_CC	cytoplasm	32	54.24	2.75E-07
GOTERM_CC	nucleoplasm	31	52.54	1.44E-06
GOTERM_CC	plasma membrane	24	40.68	4.48E-06

GOTERM_CC	mitochondrion	17	28.81	9.02E-06
GOTERM_CC	extracellular region	17	28.81	1.94E-05
GOTERM_CC	macromolecular complex	16	27.12	4.90E-05
GOTERM_CC	membrane	16	27.12	6.62E-05
GOTERM_CC	extracellular space	12	20.34	1.91E-04
GOTERM_CC	perinuclear region of cytoplasm	11	18.64	3.79E-04
GOTERM_CC	chromatin	10	16.95	6.71E-04
GOTERM_CC	transcription factor complex	9	15.25	7.17E-04
GOTERM_CC	Golgi apparatus	8	13.56	7.50E-04
GOTERM_CC	neuron projection	7	11.86	0.00116218
GOTERM_CC	intracellular membrane-bounded organelle	7	11.86	0.00190841
GOTERM_CC	membrane raft	6	10.17	0.00210921
GOTERM_CC	cell surface	6	10.17	0.00269461
GOTERM_CC	caveola	5	8.47	0.00275736
GOTERM_CC	RNA polymerase II transcription factor complex	5	8.47	0.00327898
GOTERM_CC	lamellipodium	5	8.47	0.00334459
GOTERM_CC	receptor complex	5	8.47	0.00388562
GOTERM_CC	endosome	5	8.47	0.00441229
GOTERM_MF	enzyme binding	18	30.51	1.52E-15
GOTERM_MF	transcription factor binding	12	20.34	3.42E-11
GOTERM_MF	macromolecular complex binding	14	23.73	2.92E-10
GOTERM_MF	protein binding	58	98.31	1.58E-09
GOTERM_MF	identical protein binding	23	38.98	2.39E-09
GOTERM_MF	RNA polymerase II sequence-specific DNA binding transcription factor binding	8	13.56	1.83E-06
GOTERM_MF	protein kinase binding	11	18.64	3.16E-06
GOTERM_MF	protein phosphatase binding	6	10.17	9.73E-06
GOTERM_MF	peptidase activity	6	10.17	1.53E-05
GOTERM_MF	protein homodimerization activity	11	18.64	5.83E-05
GOTERM_MF	heme binding	6	10.17	1.00E-04
GOTERM_MF	protein serine/threonine kinase activity	8	13.56	1.91E-04
GOTERM_MF	transcription cofactor binding	4	6.78	2.15E-04
GOTERM_MF	cytokine activity	6	10.17	3.06E-04
GOTERM_MF	cysteine-type endopeptidase activity involved in execution phase of apoptosis	3	5.08	3.30E-04
GOTERM_MF	ubiquitin protein ligase binding	7	11.86	3.45E-04
GOTERM_MF	protease binding	5	8.47	3.77E-04
GOTERM_MF	cysteine-type endopeptidase activity involved in apoptotic signaling pathway	3	5.08	4.11E-04
GOTERM_MF	cysteine-type endopeptidase activity involved in apoptotic process	3	5.08	4.11E-04
GOTERM_MF	transcription regulatory region sequence-specific DNA binding	6	10.17	8.29E-04
GOTERM_MF	MAP kinase activity	3	5.08	0.00108388
GOTERM_MF	MAP kinase kinase activity	3	5.08	0.0013765

GOTERM_MF	ATP binding	13	22.03	0.0022478
GOTERM_MF	R-SMAD binding	3	5.08	0.00245388
GOTERM_MF	cyclin binding	3	5.08	0.0048905

Table S5. KEGG enrichment analysis results.

Category	Term	Count	%	P Value
KEGG_PATHWAY	Pathways in cancer	34	57.63	9.49E-25
KEGG_PATHWAY	Kaposi sarcoma-associated herpesvirus infection	21	35.59	9.93E-19
KEGG_PATHWAY	Human cytomegalovirus infection	21	35.59	1.92E-17
KEGG_PATHWAY	Hepatitis B	20	33.90	7.29E-19
KEGG_PATHWAY	Proteoglycans in cancer	20	33.90	6.56E-17
KEGG_PATHWAY	Lipid and atherosclerosis	20	33.90	1.61E-16
KEGG_PATHWAY	Human papillomavirus infection	20	33.90	4.78E-13
KEGG_PATHWAY	PI3K-Akt signaling pathway	20	33.90	1.59E-12
KEGG_PATHWAY	Hepatitis C	18	30.51	2.70E-16
KEGG_PATHWAY	Salmonella infection	18	30.51	6.42E-13
KEGG_PATHWAY	MicroRNAs in cancer	18	30.51	2.21E-11
KEGG_PATHWAY	AGE-RAGE signaling pathway in diabetic complications	17	28.81	3.79E-18
KEGG_PATHWAY	Epstein-Barr virus infection	17	28.81	3.49E-13
KEGG_PATHWAY	Prostate cancer	16	27.12	8.27E-17
KEGG_PATHWAY	Chagas disease	16	27.12	1.81E-16
KEGG_PATHWAY	Gastric cancer	16	27.12	6.19E-14
KEGG_PATHWAY	Tuberculosis	16	27.12	1.03E-12
KEGG_PATHWAY	Chemical carcinogenesis - receptor activation	16	27.12	1.11E-11
KEGG_PATHWAY	MAPK signaling pathway	16	27.12	1.15E-09
KEGG_PATHWAY	Pathways of neurodegeneration - multiple diseases	16	27.12	7.13E-07
KEGG_PATHWAY	Colorectal cancer	15	25.42	4.70E-16
KEGG_PATHWAY	Small cell lung cancer	15	25.42	1.26E-15
KEGG_PATHWAY	HIF-1 signaling pathway	15	25.42	1.52E-14
KEGG_PATHWAY	Toxoplasmosis	15	25.42	2.23E-14
KEGG_PATHWAY	FoxO signaling pathway	15	25.42	2.07E-13
KEGG_PATHWAY	Measles	15	25.42	4.75E-13
KEGG_PATHWAY	Focal adhesion	15	25.42	7.48E-11
KEGG_PATHWAY	Alzheimer disease	15	25.42	3.13E-07
KEGG_PATHWAY	IL-17 signaling pathway	14	23.73	5.50E-14
KEGG_PATHWAY	Yersinia infection	14	23.73	7.81E-12
KEGG_PATHWAY	Cellular senescence	14	23.73	4.12E-11
KEGG_PATHWAY	Influenza A	14	23.73	1.32E-10
KEGG_PATHWAY	Pathogenic Escherichia coli infection	14	23.73	7.71E-10
KEGG_PATHWAY	Chemical carcinogenesis - reactive oxygen species	14	23.73	3.54E-09

KEGG_PATHWAY	Shigellosis	14	23.73	1.22E-08
KEGG_PATHWAY	Endometrial cancer	13	22.03	3.49E-15
KEGG_PATHWAY	Pertussis	13	22.03	1.09E-13
KEGG_PATHWAY	Pancreatic cancer	13	22.03	1.09E-13
KEGG_PATHWAY	PD-L1 expression and PD-1 checkpoint pathway in cancer	13	22.03	7.79E-13
KEGG_PATHWAY	C-type lectin receptor signaling pathway	13	22.03	5.24E-12
KEGG_PATHWAY	TNF signaling pathway	13	22.03	1.28E-11
KEGG_PATHWAY	Apoptosis	13	22.03	1.30E-10
KEGG_PATHWAY	Fluid shear stress and atherosclerosis	13	22.03	1.68E-10
KEGG_PATHWAY	Breast cancer	13	22.03	3.24E-10
KEGG_PATHWAY	Viral carcinogenesis	13	22.03	1.39E-08
KEGG_PATHWAY	Rap1 signaling pathway	13	22.03	1.93E-08
KEGG_PATHWAY	Human immunodeficiency virus 1 infection	13	22.03	2.15E-08
KEGG_PATHWAY	Human T-cell leukemia virus 1 infection	13	22.03	3.59E-08
KEGG_PATHWAY	Coronavirus disease - COVID-19	13	22.03	5.86E-08
KEGG_PATHWAY	Leishmaniasis	12	20.34	4.01E-12
KEGG_PATHWAY	EGFR tyrosine kinase inhibitor resistance	12	20.34	5.37E-12
KEGG_PATHWAY	Endocrine resistance	12	20.34	6.01E-11
KEGG_PATHWAY	Toll-like receptor signaling pathway	12	20.34	1.16E-10
KEGG_PATHWAY	Non-alcoholic fatty liver disease	12	20.34	8.55E-09
KEGG_PATHWAY	JAK-STAT signaling pathway	12	20.34	1.36E-08
KEGG_PATHWAY	Hepatocellular carcinoma	12	20.34	1.99E-08
KEGG_PATHWAY	Bladder cancer	11	18.64	1.37E-13
KEGG_PATHWAY	VEGF signaling pathway	11	18.64	6.99E-12
KEGG_PATHWAY	Non-small cell lung cancer	11	18.64	5.56E-11
KEGG_PATHWAY	Melanoma	11	18.64	5.56E-11
KEGG_PATHWAY	T cell receptor signaling pathway	11	18.64	2.28E-09
KEGG_PATHWAY	Thyroid hormone signaling pathway	11	18.64	1.01E-08
KEGG_PATHWAY	Osteoclast differentiation	11	18.64	1.74E-08
KEGG_PATHWAY	Alcoholic liver disease	11	18.64	4.72E-08
KEGG_PATHWAY	NOD-like receptor signaling pathway	11	18.64	5.41E-07
KEGG_PATHWAY	Herpes simplex virus 1 infection	11	18.64	0.00229347
KEGG_PATHWAY	Platinum drug resistance	10	16.95	1.65E-09
KEGG_PATHWAY	Glioma	10	16.95	2.11E-09
KEGG_PATHWAY	Chronic myeloid leukemia	10	16.95	2.39E-09
KEGG_PATHWAY	Amoebiasis	10	16.95	3.33E-08
KEGG_PATHWAY	Sphingolipid signaling pathway	10	16.95	1.28E-07
KEGG_PATHWAY	Neurotrophin signaling pathway	10	16.95	1.28E-07
KEGG_PATHWAY	Acute myeloid leukemia	9	15.25	1.86E-08
KEGG_PATHWAY	Renal cell carcinoma	9	15.25	2.35E-08

KEGG_PATHWAY	Central carbon metabolism in cancer	9	15.25	2.64E-08
KEGG_PATHWAY	p53 signaling pathway	9	15.25	3.70E-08
KEGG_PATHWAY	ErbB signaling pathway	9	15.25	1.24E-07
KEGG_PATHWAY	Rheumatoid arthritis	9	15.25	2.51E-07
KEGG_PATHWAY	Th17 cell differentiation	9	15.25	8.00E-07
KEGG_PATHWAY	Relaxin signaling pathway	9	15.25	3.09E-06
KEGG_PATHWAY	Autophagy - animal	9	15.25	6.00E-06
KEGG_PATHWAY	Signaling pathways regulating pluripotency of stem cells	9	15.25	6.66E-06
KEGG_PATHWAY	Ras signaling pathway	9	15.25	2.38E-04
KEGG_PATHWAY	Prion disease	9	15.25	6.33E-04
KEGG_PATHWAY	Thyroid cancer	8	13.56	5.49E-09
KEGG_PATHWAY	Legionellosis	8	13.56	1.26E-07
KEGG_PATHWAY	Inflammatory bowel disease	8	13.56	3.18E-07
KEGG_PATHWAY	Prolactin signaling pathway	8	13.56	5.33E-07
KEGG_PATHWAY	Choline metabolism in cancer	8	13.56	5.28E-06
KEGG_PATHWAY	mTOR signaling pathway	8	13.56	1.08E-04
KEGG_PATHWAY	Necroptosis	8	13.56	1.22E-04
KEGG_PATHWAY	Axon guidance	8	13.56	2.81E-04
KEGG_PATHWAY	Neutrophil extracellular trap formation	8	13.56	3.65E-04
KEGG_PATHWAY	Chemokine signaling pathway	8	13.56	3.89E-04
KEGG_PATHWAY	Transcriptional misregulation in cancer	8	13.56	4.01E-04
KEGG_PATHWAY	Malaria	7	11.86	1.23E-06
KEGG_PATHWAY	Fc epsilon RI signaling pathway	7	11.86	7.69E-06
KEGG_PATHWAY	Progesterone-mediated oocyte maturation	7	11.86	7.85E-05
KEGG_PATHWAY	NF-kappa B signaling pathway	7	11.86	8.75E-05
KEGG_PATHWAY	Growth hormone synthesis, secretion and action	7	11.86	1.93E-04
KEGG_PATHWAY	Natural killer cell mediated cytotoxicity	7	11.86	2.52E-04
KEGG_PATHWAY	Estrogen signaling pathway	7	11.86	4.11E-04
KEGG_PATHWAY	Apelin signaling pathway	7	11.86	4.28E-04
KEGG_PATHWAY	Phospholipase D signaling pathway	7	11.86	5.97E-04
KEGG_PATHWAY	Cushing syndrome	7	11.86	7.62E-04
KEGG_PATHWAY	Regulation of actin cytoskeleton	7	11.86	0.00430328
KEGG_PATHWAY	cAMP signaling pathway	7	11.86	0.00459969
KEGG_PATHWAY	B cell receptor signaling pathway	6	10.17	2.73E-04
KEGG_PATHWAY	GnRH signaling pathway	6	10.17	4.90E-04
KEGG_PATHWAY	Fc gamma R-mediated phagocytosis	6	10.17	5.95E-04
KEGG_PATHWAY	Insulin resistance	6	10.17	9.69E-04
KEGG_PATHWAY	Cholinergic synapse	6	10.17	0.00118778
KEGG_PATHWAY	Platelet activation	6	10.17	0.0017956
KEGG_PATHWAY	Cell cycle	6	10.17	0.00192684

KEGG_PATHWAY	Oxytocin signaling pathway	6	10.17	0.00458772
KEGG_PATHWAY	African trypanosomiasis	5	8.47	1.27E-04
KEGG_PATHWAY	GnRH secretion	5	8.47	0.00106118
KEGG_PATHWAY	Epithelial cell signaling in Helicobacter pylori infection	5	8.47	0.00148375
KEGG_PATHWAY	Adherens junction	5	8.47	0.00156408
KEGG_PATHWAY	Bacterial invasion of epithelial cells	5	8.47	0.00211075
KEGG_PATHWAY	Longevity regulating pathway	5	8.47	0.00357567
KEGG_PATHWAY	Th1 and Th2 cell differentiation	5	8.47	0.00402804
KEGG_PATHWAY	TGF-beta signaling pathway	5	8.47	0.00435028
KEGG_PATHWAY	Apoptosis - multiple species	4	6.78	0.00145822
KEGG_PATHWAY	Ferroptosis	4	6.78	0.0029954
KEGG_PATHWAY	Graft-versus-host disease	4	6.78	0.00320966
KEGG_PATHWAY	Type II diabetes mellitus	4	6.78	0.00415984
